# High-resolution crystal structure of the therapeutic antibody pembrolizumab bound to the human PD-1

**DOI:** 10.1038/srep35297

**Published:** 2016-10-13

**Authors:** Shoichiro Horita, Yayoi Nomura, Yumi Sato, Tatsuro Shimamura, So Iwata, Norimichi Nomura

**Affiliations:** 1 Department of Cell Biology, Graduate School of Medicine, Kyoto University, Yoshida-Konoe-cho, Sakyo-ku, Kyoto 606-8501, Japan; 2 Japan Science and Technology Agency, Research Acceleration Program, Membrane Protein Crystallography Project, Yoshida-Konoe-cho, Sakyo-ku, Kyoto 606-8501, Japan.; 3 RIKEN SPring-8 Center, Kouto, Sayo-cho, Sayo-gun, Hyogo 679-5148, Japan

## Abstract

Pembrolizumab is an FDA-approved therapeutic antibody that targets the programmed cell death-1 (PD-1) to block the immune checkpoint pathway for the treatment of various types of cancer. It receives remarkable attention due to the high degree of efficacy. Very recently, the crystal structure of the Fab fragment of pembrolizumab (PemFab) in complex with the extracellular domain of human PD-1 (PD-1_ECD_) was reported at a resolution of 2.9 Å. However, this relatively low-resolution structural data fails to provide sufficient information on interfacial water molecules at the binding interface that substantially contribute to affinity and specificity between the therapeutic antibody and target. Here, we present the independently determined crystal structure of the Fv fragment of pembrolizumab (PemFv) in complex with the PD-1_ECD_ at a resolution of 2.15 Å. This high-resolution structure allows the accurate mapping of the interaction including water-mediated hydrogen bonds and provides, for the first time, a coherent explanation of PD-1 antagonism by pembrolizumab. Our structural data also provides new insights into the rational design of improved anti-PD-1 therapeutics.

When the PD-1 (also known as CD279) inhibitory receptor binds its endogenous ligand, PD-L1 (CD274, B7-H1), the resulting signalling suppresses immune responses against autoantigens and plays an important role in the maintenance of peripheral immune tolerance^1^. However, a significantly increased expression of PD-L1 in various tumours permits these malignant cells to escape destruction by the immune system^2^,^3^. The PD-1/PD-L1 interaction inhibits T-lymphocyte proliferation, release of cytokines, and cytotoxicity, resulting in exhaustion of tumour-specific T cells^4^. The blockage of the PD-1/PD-L1 pathway results in the reversal of the exhausted T-cell phenotype and the normalization of the anti-tumour response, providing a rationale for cancer immunotherapy^5^.

Targeting the PD-1/PD-L1 interaction with monoclonal antibodies has demonstrated great promise as a strategy for controlling and eradicating cancer. Two antibodies against PD-1, pembrolizumab (Keytruda, Merck and Co.) and nivolumab (Opdivo, Bristol-Myers Squibb), were approved by the U.S. Food and Drug Administration (FDA) in 2014 for patients with advanced melanoma^6^,^7^. Recent clinical trials have shown that these antibodies are effective against other cancers, such as non-small cell lung cancer, renal cell carcinoma, bladder cancer, and Hodgkin’s lymphoma^8^. It is widely expected that anti-PD-1 antibodies are likely to become an important component of treatment for a variety of malignancies. Although these antibodies are associated with substantial benefits, the immune checkpoint blockade can lead to inflammatory side effects^9^. Obtaining the atomic structure of the human PD-1/therapeutic antibody complex is essential for understanding its inhibition mechanism and the design of improved anti-PD-1 therapeutics. Very recently, the crystal structure of the Fab fragment of pembrolizumab in complex with the extracellular domain of human PD-1 (PD-1_ECD_) has been determined at a resolution of 2.9 Å^10^. Although the binding site of pembrolizumab on PD-1 has been roughly identified, this relatively low-resolution structural data does not provide sufficient information on interfacial water molecules at the binding interface that substantially contribute to affinity and specificity between the receptor and therapeutic antibody. To provide a sufficient rationale for PD-1 antagonism by pembrolizumab, it is necessary to visualize water-mediated hydrogen bonds with higher-resolution structural data.

Herein, we present the independently determined crystal structure of the Fv fragment of pembrolizumab (PemFv) in complex with PD-1_ECD_ at a resolution of 2.15 Å and compare its intermolecular interface with that of the PD-L1/PD-1_ECD_ complex including water-mediated hydrogen bond networks. Our high-resolution structural data provides a coherent explanation of the mode of competitive inhibitory action by pembrolizumab. Moreover, it provides new insights into the rational design of improved anti-PD-1 therapeutics.

## Results and Discussion

### Structure of pembrolizumab Fv in complex with PD-1

Considering that both PemFv and PD-1_ECD_ contain intrachain disulfide bonds, a Gram-positive bacterial secretion expression system was used to produce these proteins for crystallography (Methods). The resulting co-crystals appeared in the space group *P*2_1_2_1_2_1_, and the structure was determined by molecular replacement to a resolution of 2.15 Å (*R*_work_ and *R*_free_ values of 18.4% and 22.6%, respectively)(Table 1 and Supplementary Figure S1). Although each asymmetric unit of the crystals contains four complex assemblies, analytical gel filtration confirmed that the 1:1 complex (approximately 42 kDa) exists as a monomer in solution (data not shown).

**Figure 1:**
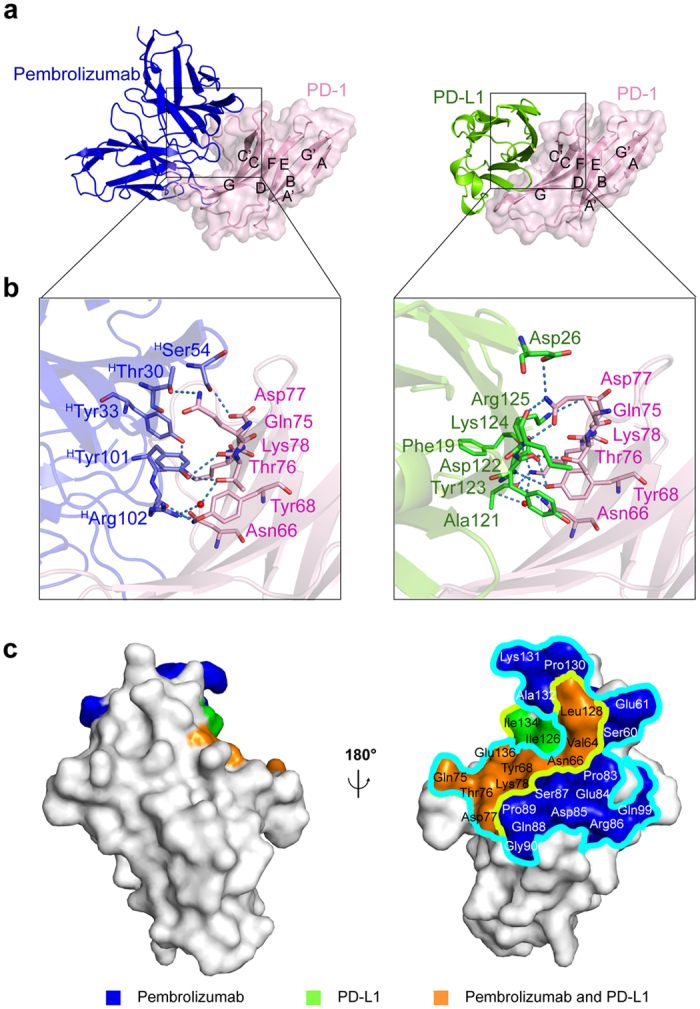
Structure of the pembrolizumab/PD-1 complex and comparison with the PD-L1/PD-1 complex. (**a**) Overall structures of the PemFv/human PD-1_ECD_ complex (this study; left) and the human PD-L1_ECD-N_/human PD-1_ECD_ complex (PDB ID: 4ZQK; right). PD-1_ECD_, PemFv and PD-L1_ECD-N_ are shown in light pink (surface representation), blue (ribbon diagram) and green (ribbon diagram), respectively. Canonical designations of β strands within PD-1_ECD_ are also shown. (**b**) Close-up views of interfaces. Residues involved in hydrogen bonds (blue dashes) are shown. The color-coding is the same as in (**a**). A water molecule is shown in red. (**c**) Steric overlap on the PD-1 surface that interact with pembrolizumab and PD-L1. The pembrolizumab epitope (outlined in light blue) overlaps with the binding regions for PD-L1 (outlined in light green). PD-1_ECD_ is represented by a grey surface and rotated by 180° around the vertical axis. The binding regions and the overlapping areas are coloured and marked differently.

**Figure 2:**
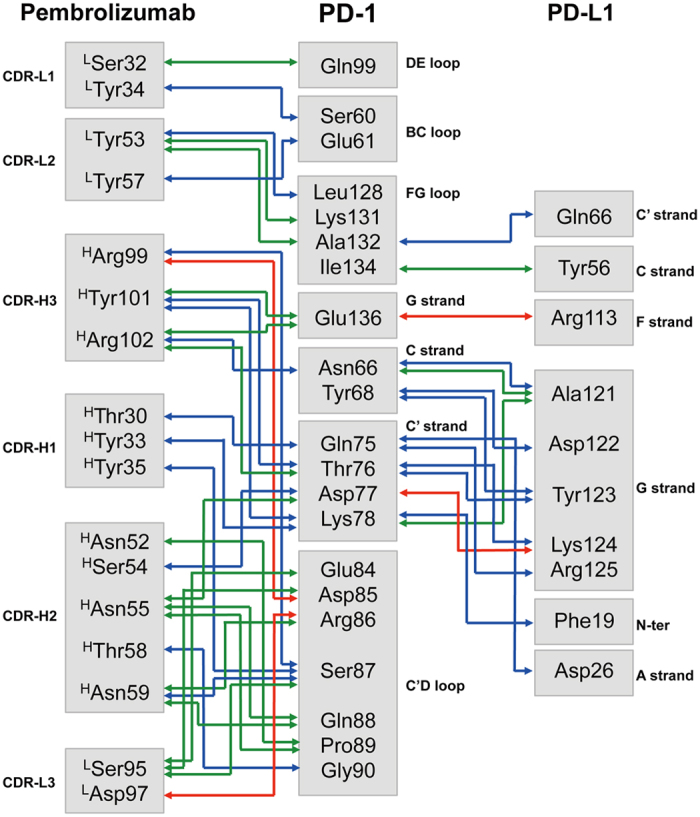
A schematic diagram of polar interactions. Direct protein/protein hydrogen bonds are in blue; water-mediated hydrogen bonds are in green; and salt bridges are in red.

The overall structure of the PemFv/PD-1_ECD_ complex shows that the complementarity determining regions (CDRs) of PemFv interact predominantly with a major groove in PD-1_ECD,_ which is formed on one surface by the CC’FG antiparallel β−sheet and the BC, C’D, and FG loops (Fig. 1a). The interface includes 15 direct hydrogen bonds between residues, 15 water-mediated hydrogen bonds, two salt bridges, and hydrophobic interactions, and a total of 26 residues of PD-1_ECD_ are involved in the interaction (Fig. 2). The complex formation buries a very large solvent-accessible surface area of 1,137 Å^2^ on PD-1_ECD_, which is much higher than the common value observed in other antigen/antibody complexes^11^. Most prominently, residues located in loop C’D (Pro84 to Gly90) and in strand C’ (Gln75 to Lys78) of PD-1_ECD_ provide a key part of the interactions, mainly through hydrogen bonds and salt bridges to the CDR-L3, CDR-H1, CDR-H2 and CDR-H3 of pembrolizumab. The N-linked glycosylated residues Asn49, Asn58, Asn74 and Asn116 are located away from the interface, suggesting that the sugar chains have no direct contact with pembrolizumab.

Less surprisingly, the overall structure of the PemFv/PD-1_ECD_ complex reported here was similar to that of the PemFab/PD-1_ECD_ complex (PDB ID: 5JXE)^10^. Both structure’s backbones align very well with a root-mean-square deviation (r.m.s.d.) of 0.38 Å (with 192 Cα atoms). Nevertheless, one important novelty of our study is the visualization of 15 water-mediated hydrogen bonds at the binding interface (Fig. 2, green lines). The previously reported PemFab/PD-1_ECD_ complex structure only visualizes 8 direct hydrogen bonds and one salt bridge. Because of its relatively low resolution of 2.9 Å, there is no assignment of hydrogen-bonded interfacial water molecules that mediate imperfect surface complementarity of the antibody/antigen interface (Supplementary Figure S2). Without considering the structural and energetic contribution of the interfacial water molecules, one cannot provide a sufficient rationale for the extremely high binding affinity of pembrolizumab against PD-1.

### Comparison of binding sites of PD-1 for pembrolizumab and PD-L1

It has been shown that the intermolecular interaction between PD-1 and PD-L1 occurs mainly via the CC’FG sheet within both proteins^12^,^13^. In the crystal structure of human PD-1_ECD_ in complex with the N-terminal half of the extracellular domain of human PD-L1 (PD-L1_ECD-N_) (PDB ID: 4ZQK)^13^, there is a hydrophobic surface patch formed by residues Val64, Tyr68, Ile126, Leu128, Ala132 and Ile134 in PD-1_ECD_ that interacts with PD-L1_ECD-N_. Surrounding the hydrophobic patch, several hydrophilic residues of PD-L1_ECD-N_ interact with residues Asn66, Tyr68, Gln75, Thr76, Asp77, Lys78, Ala132 and Glu136 of PD-1_ECD_, forming numerous hydrogen bonds and salt bridges (Fig. 1b and Fig. 2). In total, 12 residues of PD-1_ECD_ participate in its interactions with PD-L1_ECD-N_, forming 9 direct hydrogen bonds between residues, three water-mediated hydrogen bonds, two salt bridges and hydrophobic interactions. The interface buries 796 Å^2^ of the solvent-accessible surface area on PD-1_ECD_.

A structural comparison between the PemFv/PD-1_ECD_ complex and the PD-L1_ECD-N_/PD-1_ECD_ complex indicates that the epitope recognized by pembrolizumab overlaps largely with the residues in PD-1 responsible for the interaction with PD-L1. Seven residues (Asn66, Gln75, Thr76, Asp77, Lys78, Ala132 and Glu136) of PD-1_ECD_ participate in polar interactions with both PemFv and PD-L1_ECD-N_, and a solvent-accessible surface area of 682 Å^2^ on PD-1_ECD_ is commonly buried in the complex with either PemFv or PD-L1_ECD-N_ (Fig. 1c and Fig. 2). The disparity of binding affinities (a *K*_D_ value of 8.2 μM for PD-1_ECD_/PD-L1_ECD-N_^14^, versus a *K*_D_ value of 27 pM for PD-1_ECD_/pembrolizumab IgG^15^) can be attributed to a significant difference in the number of polar interactions, including water-mediated hydrogen bonds. These observations rationalize that the binding of pembrolizumab to PD-1 would compete with the binding of PD-L1 to the receptor. In the absence of pembrolizumab, the binding of the inactive PD-1 receptor to the PD-L1 dimer^16^ would trigger receptor dimerization, followed by the *trans*-phosphorylation of a tyrosine within the immunoreceptor tyrosine-based switch motif (ITSM), the recruitment of the protein tyrosine phosphatase SHP-2, and the dephosphorylation of the T-cell receptor (TCR) proximal signalling molecules including ZAP70, PKC*θ*, and CD3*ζ*. This would lead to the attenuation of the TCR signal to suppress the immune response^17^. However, in the presence of a sufficient amount of pembrolizumab, the PD-L1 binding site on PD-1 would be blocked, and thus, PD-1 would not be able to undergo dimerization. Therefore, the PD-1 signalling would not be performed.

### Implications for development of improved anti-PD-1 drugs

**Figure 3:**
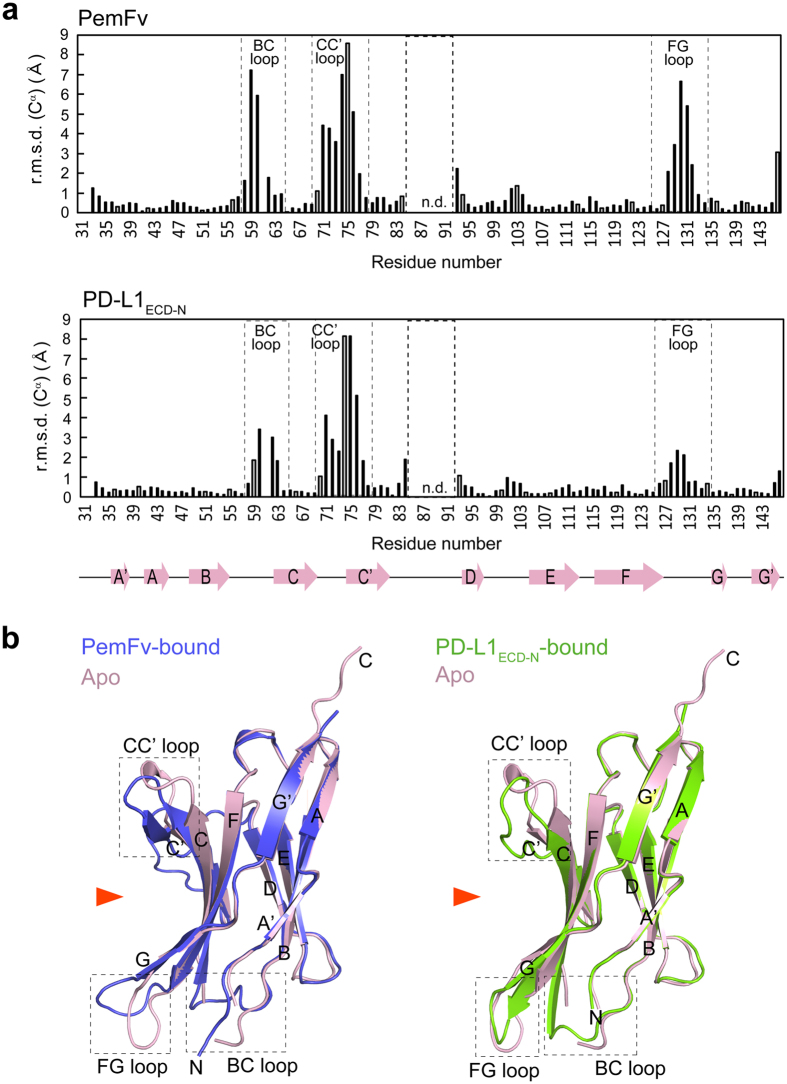
Conformational changes of PD-1 upon binding of pembrolizumab or PD-L1. (**a**) The r.m.s.d. for Cα atoms between the pair of PD-1_ECD_ in apo-form and PemFv-bound form (upper panel), and between the pair of PD-1_ECD_ in apo-form and PD-L1-bound form (lower panel). The secondary structural features of PD-1_ECD_ are depicted at the bottom. Boxes in a dotted line highlight loops experiencing major conformational changes. n.d.: not determined. (**b**) Superposition of PD-1_ECD_ molecules. PD-1_ECD_ in apo- and PemFv-bound form (left), and PD-1_ECD_ in apo- and PD-L1_ECD-N_-bound form (right). Orange arrows represent interfaces with which PemFv and PD-L1_ECD-N_ interact.

Pembrolizumab forms a large, flat paratope to accommodate a large conformational epitope on PD-1. Upon the binding of pembrolizumab, PD-1_ECD_ undergoes a peripheral conformational change, probably because of an induced fit, to generate a “crescent”-like structure with a shallow concave groove; this differs from the flat surfaces of the apo- and PD-L1-bound forms of PD-1_ECD_ (Fig. 3). However, the groove is too shallow to design traditional small-molecule drugs that can bind tightly to prevent the binding of PD-L1.

**Figure 4:**
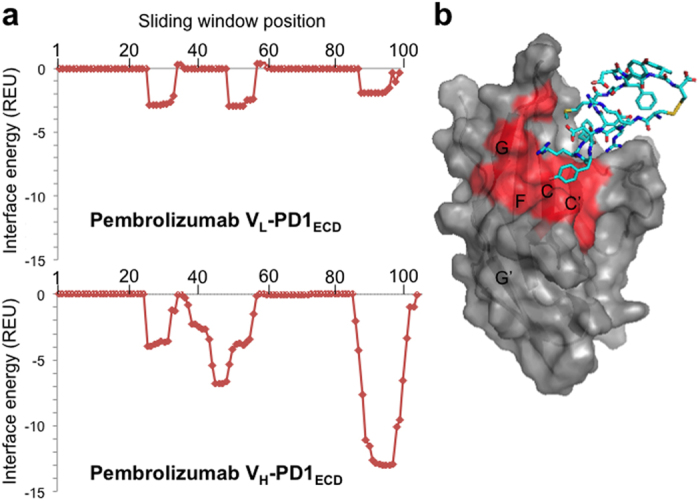
Structure-based identification of “hot segments” in the pembrolizumab-PD-1 interaction and design of a putative cyclic peptide binder. (**a**) An output of Peptiderive program^18^. Intermolecular binding energies between PD-1_ECD_ and all overlapping 13-mer peptide segments derived from the two variable domains (V_L_ and V_H_) of pembrolizumab were calculated on the basis of the structural data of the PemFv/PD-1_ECD_ complex, as reported in REU (Rosetta energy units). The horizontal axis indicates the starting residue of each sliding window consisting of a 13-mer peptide segment. (**b**) A structural model of the complex of PD-1_ECD_ (surface) and a cyclic peptide (stick representation) with a favourable binding energy. The residues of PD-1_ECD_ that are involved in the interactions with both pembrolizumab and PD-L1 are coloured in red. Canonical designations of β strands are also shown.

Instead, the structural data reported here is a useful resource for the rational design of middle-sized molecules such as cyclic peptides or DNA and RNA aptamers that may possibly more easily mimic the effect of the binding of pembrolizumab to PD-1. For instance, on the basis of the structural data of the PemFv/PD-1_ECD_ complex, the Rosetta Peptiderive program^18^ identified that a 13-residue linear polypeptide segment spanning from ^H^Ala97 to ^H^Tyr109 of PemFv contributes most predominantly to the complex binding energy (Fig. 4a). A cyclic peptide derivative of this “hot segment” (Cys-Ala-Arg-Arg-Asp-Tyr-Arg-Phe-Asp-Met-Gly-Phe-Asp-Tyr-Trp-Gly-Cys; a peptide structurally constrained by a disulfide bond between the terminal Cys residues) was designed, and the complex formation with PD-1_ECD_ was modelled (Fig. 4b). In this model, the binding site of the cyclic peptidomimetic is overlapped with that of PD-L1. The PRODIGY program^19^ estimated that a K_D_ value of the binding affinity of the cyclic peptide to PD-1_ECD_ is 5.6 × 10^−7^ M. This cyclic peptide would be a good starting material for the development of downsized blockers of the PD-1/PD-L1 pathway. Structure-guided design of such peptidomimetics could overcome disadvantages of the current therapeutic antibody associated with the extremely high cost of commercial-scale production and the limited penetration into tissues.

Our high-resolution structural data of the PemFv/PD-1_ECD_ complex also serves as a guide for further engineering of pembrolizumab to strike a balance between its efficacy and reducing its adverse effects. Different combinations of targeted disruption of the direct or water-mediated hydrogen bonds and the salt bridges observed at the binding interface should modulate the affinity of pembrolizumab against PD-1. Such structure-guided mutagenesis can generate derivatives with lower affinities for PD-1, resulting in a shift of the binding equilibrium towards partially active (PD-L1-bound) states of PD-1. Creating partially inverse agonist antibodies would have novel implications for immune cell signalling and its regulation.

In summary, we report the high-resolution structure of pembrolizumab Fv in complex with the human PD-1 extracellular domain, which enables us to identify the detailed intermolecular interface between pembrolizumab and PD-1. This structure allows for a deep understanding and mechanistic interpretation of PD-1 immune blockade therapy by pembrolizumab. It also provides a good starting point for facilitating efforts to develop improved anti-PD-1 therapeutics to modulate immune responses to fight cancer.

## Methods

### Complete amino-acid sequence of the expression constructs

The cloned *Homo sapiens* PD-1 sequence contains residues 32 to 160 from the complete 288 residues (UniProt accession number: Q15116); The C93S mutation is underlined, and additional N- and C-terminal residues retained after restriction site cloning or TEV cleavage are shown in italics (refer to the following section for cloning details):

*GS*WNPPTFSPALLVVTEGDNATFTCSFSNTSESFVLNWYRMSPSNQTDKLAAFPEDRSQPGQDSRFRVTQLPNGRDFHMSVVRARRNDSGTYLCGAISLAPKAQIKESLRAELRVTERRAEVPTAHPSPSP*TSENLYFQ.*

The pembrolizumab light chain variable region (PemV_L_); the additional C-terminal residues that were retained after TEV cleavage are shown in italics:

EIVLTQSPATLSLSPGERATLSCRASKGVSTSGYSYLHWYQQKPGQAPRLLIYLASYLESGVPARFSGSGSGTDFTLTISSLEPEDFAVYYCQHSRDLPLTFGGGTKVEIK*TSENLYFQ.*

The pembrolizumab heavy chain variable region (PemV_H_):

QVQLVQSGVEVKKPGASVKVSCKASGYTFTNYYMYWVRQAPGQGLEWMGGINPSNGGTNFNEKFKNRVTLTTDSSTTTAYMELKSLQFDDTAVYYCARRDYRFDMGFDYWGQGTTVTVSS.

### Protein expression and purification

The PD-1 extracellular domain (PD-1_ECD_) and pembrolizumab Fv (PemFv) were expressed in *Brevibacillus choshinensis* and secreted as His_6_-tagged proteins. The proteins were purified from culture medium. The artificially synthesized codon-optimized cDNA of PD-1_ECD_, PemV_L_ and PemV_H_ were inserted downstream of and in frame with the secretion signal sequence of the plasmid pNY326 (Clontech), which contains a neomycin-resistance gene and the constitutively active promoter P5. To facilitate the detection and purification of the secreted proteins, the sequences for the tobacco etch virus (TEV) protease cleavage site and a His_6_ tag were placed at the C-termini of the PD-1_ECD_ and PemV_L_ cDNAs. All cloned inserts were verified by sequencing of both strands. Non-sporulating *Brevibacillus choshinensis* HPD31-SP3 cells (Clontech) were electroporated with the individual plasmids under the conditions of 7.5 kV/cm, 25 μF, and 1000 Ω according to the manufacturer’s instructions. The cells were grown at 30 °C and 200 rpm in 2SY medium (soypton 40 g/L, yeast extract 5 g/L, glucose 20 g/L, and CalCl_2_ 0.15 g/L) supplemented with 50 mg/L neomycin. For the expression of PemFv, the cells expressing PemV_L_ and PemV_H_ were initially grown separately as overnight precultures. The precultures were then combined, diluted in 2SY medium to give an OD_600_ of 0.02 for each strain, and grown for 65–70 h. The cells were removed by centrifugation at 6,000 *g* for 15 min. The recovered culture supernatant was adjusted to a final ammonium sulfate concentration of 60% saturation. The precipitate was pelleted, dissolved in TBS buffer (10 mM Tris-HCl, pH 7.5, 150 mM NaCl), and dialyzed overnight against the same buffer. The dialyzed sample was mixed with Ni-NTA resin equilibrated with buffer A (10 mM Tris-HCl, pH 7.5, 150 mM NaCl, and 20 mM imidazole). Bound proteins were eluted with buffer B (10 mM Tris-HCl, pH 7.5, 150 mM NaCl, and 250 mM imidazole), mixed with TEV-His_6_ and dialyzed overnight against TBS buffer. The cleaved His_6_ tag and TEV-His_6_ were removed using a HisTrap column equilibrated with buffer A. Tag-free Fv fragments were concentrated and loaded onto a HiLoad 16/60 Superdex 75 column (GE Healthcare) equilibrated with TBS buffer. The peak fractions were pooled, concentrated, flash frozen in liquid nitrogen, and stored at −80 °C.

### Crystallisation

The protein complex was prepared by incubating PD-1_ECD_ with PemFv at a molar ratio of 1:2 for 1 h on ice. The complex was subjected to size exclusion chromatography (Superdex 200 10/300 column, GE Healthcare). Peak fractions containing the PD-1_ECD_/PemFv complex were concentrated to approximately 10 mg/mL by ultrafiltration (Millipore, MWCO 10 kDa) and used for the crystallisation experiments. The crystals of the complex used for structural determination were grown at 20 °C by sitting drop vapor diffusion. A 50-μL reservoir containing 20% PEG 3350 and 0.2 M KNO_3_ was equilibrated against a 0.4 μL drop containing a 1:1 mixture of the complex and reservoir solution. After 20 days of growth, the crystals were cryo-protected in 25% ethylene glycol in the mother liquor and then flash-frozen in liquid nitrogen.

### Data collection, structure determination and analysis

The diffraction data were collected at 100 K at the SPring-8 beamline BL41XU (Japan) using a PILATUS3 6M detector. The data were then integrated and scaled using XDS^20^. The structure of the PD-1_ECD_/PemFv complex was determined by molecular replacement with the program MR-PHASER^21^ using the atomic coordinates of the extracellular domain of human PD-1 (PDB ID: 3RRQ) and the Fv portion of pembrolizumab (PDB ID: 5DK3)^22^ as the search models. The model was further rebuilt in COOT^23^ and refined with phenix.refine^24^ (Table 1). The obtained crystals were merohedrally twinned according to phenix.xtriage^24^ and the data were refined with twin law (k, h, -l). Also, it should be noted that the data were processed with orthorhombic crystal system instead of tetragonal (despite of *a-* and *b-* axes with nearly the same length) based on refinement statistics. In the final Ramachandran plot, 97.7% and 2.3% of residues were in the favored and allowed regions, respectively. The refined structures were visualized with PyMOL (http://www.pymol.org/). The PISA server^25^ was used for identifying protein-protein interactions and estimating the solvent-accessible surface area. The Peptiderive server^18^ (http://rosie.rosettacommons.org/peptiderive) was used to identify hot segments of the PemFv/PD-1_ECD_ interaction. The hot segments are linear peptide stretch with significant binding energy compared to that of the whole protein-protein interaction. The program was run with setting the peptide lengths to derive as 5 to 20, separately. The PRODIGY server^19^ (http://milou.science.uu.nl/services/PRODIGY) was used to estimate the binding affinity of the complexes of peptidemimetics/PD-1_ECD_ on the basis of their structural models.

## Additional Information

**Accession codes:** The atomic coordinates and structure factors for PemFv/PD-1_ECD_ complex have been deposited in the Protein Data Bank (http://www.rcsb.org) under accession code 5B8C.

**How to cite this article**: Horita, S. *et al*. High-resolution crystal structure of the therapeutic antibody pembrolizumab bound to the human PD-1. *Sci. Rep.* **6**, 35297; doi: 10.1038/srep35297 (2016).

## Supplementary Material

Supplementary Information

## Figures and Tables

**Table 1:** Crystallographic data collection and refinement statistics.

Protein name	PemFv/PD-1_ECD_
**X-ray data collection**	
Source, wavelength	SPring-8 BL41XU, 1.00000 Å
Resolution (Å)^a^	45.4–2.15 (2.20-2.15)
Space group	*P*2_1_2_1_2_1_
Unit cell parameter (Å)	*a* 143.7, *b* 143.1, *c* 76.6
Unique reflections^a^	86668 (4231)
Redundancy^a^	6.5 (6.5)
Completeness^a^	99.6 (98.3)
*R*_merge_ (%)^a^	9.4 (111.2)
CC_1/2_ (%)^a^	99.9 (54.2)
<*I*/σ(*I*)>^a^	15.5 (1.5)
**Refinement**	
Resolution (Å)^a^	45.4–2.15 (2.18-2.15)
*R*_work_/*R*_free_ (%)^a^,^b^	18.4/22.6 (30.7/34.5)
R.m.s. deviations	
Bonds (Å)	0.004
Angles (°)	0.730
No. of atoms (average B-factors (Å^2^))	
PD-1	3519 (54.2)
Pembrolizumab	7072 (43.1)
Water	271 (46.1)
**Ramachandran plot**	
Favored region (%)	97.7
Allowed region (%)	2.3

^*a*^Values for the highest resolution shells are shown in parentheses.

^*b*^*R*_work_ was calculated with 95% of the unique reflections used for refinement, whereas *R*_free_ was calculated with the remaining 5% of the unique reflections.
