# Supplementary material for: High-resolution crystal structure of the therapeutic antibody pembrolizumab bound to the human PD-1
Source: Sci Rep. 2016 Oct 13;6:35297. doi: 10.1038/srep35297 (PMC5062252; doi:10.1038/srep35297)
Supplement: Supplementary Information [file srep35297-s1.pdf]

Supplementary Infromation

High-resolution crystal structure of the therapeutic antibody pembrolizumab bound to the human PD-1

Shoichiro Horita, Yayoi Nomura, Yumi Sato, Tatsuro Shimamura, So Iwata, and Norimichi Nomura

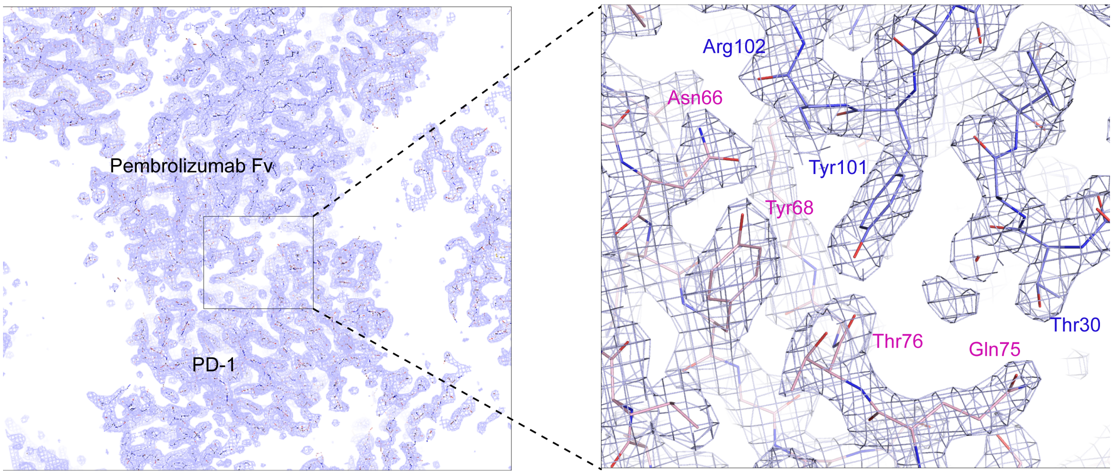

Supplementary Figure S1. Representative portions of the 2*Fo-Fc* electron density map (1.2σ). Left: PemFv/PD-1_{ECD} overall model, right: a close-up of the interface. Residues of PD-1_{ECD} and PemV_H are numbered in light pink and blue, respectively.

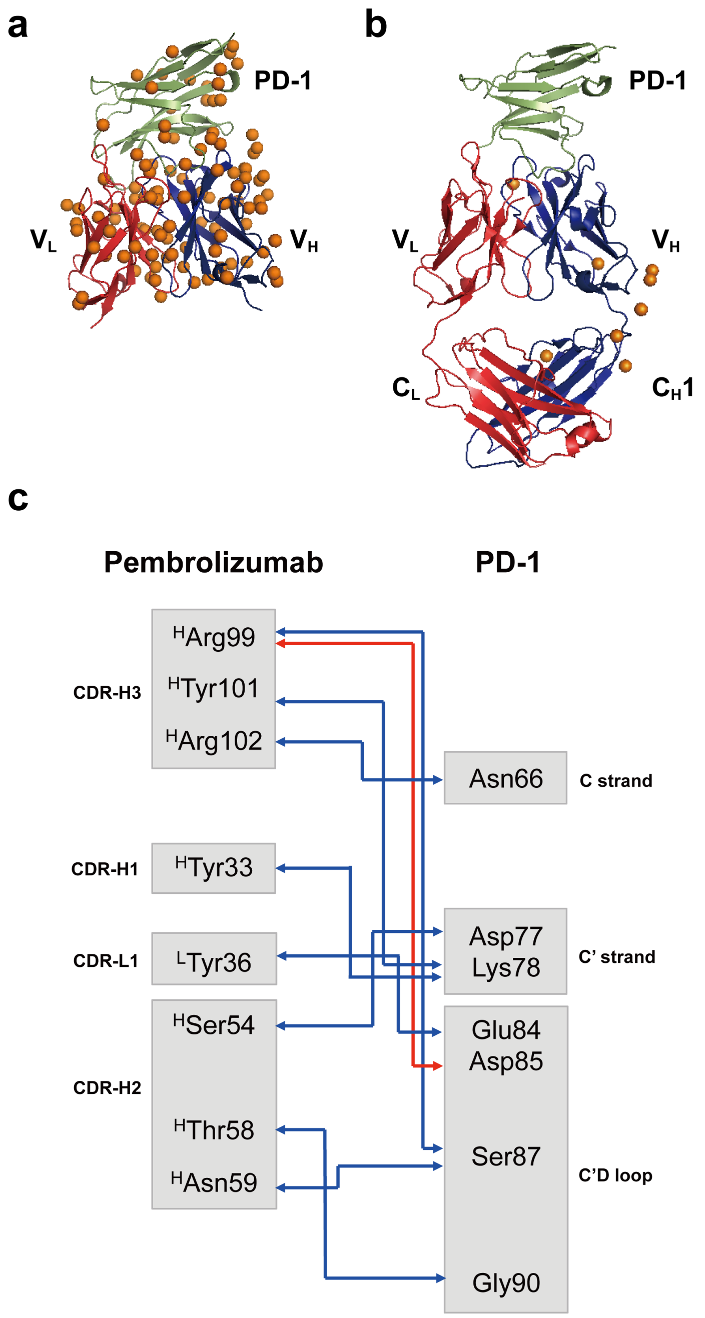

Supplementary Figure S2. Difference in quality between the two structural data, the PemFv/PD-1$_{ECD}$ complex at 2.15-Å resolution (this study) and the PemFab/ PD-1$_{ECD}$ complex at 2.9-Å resolution (Na *et al.*, 2016)[10]. (a) Position of water molecules on the surface of PemFv/PD-1$_{ECD}$ complex at a 2.15-Å resolution. Hydrogen-bonded water molecules are orange, PD-1$_{ECD}$ is green, the pembrolizumab light chain is red, and the pembrolizumab heavy chain is blue. (b) Position of water molecules on the surface of PemFab/PD-1$_{ECD}$ complex at a 2.9-Å resolution. The same colour code is used as in (a). (c) A schematic diagram of polar interactions observed in the structural data of PemFab/PD-1$_{ECD}$ complex at a 2.9-Å resolution. Direct protein/protein hydrogen bonds are blue; salt bridges are red. Water-mediated hydrogen bonds are not observed at this resolution.
